# The Metabolic Landscape in Osteoarthritis

**DOI:** 10.14336/AD.2021.1228

**Published:** 2022-07-11

**Authors:** Xiaoxin Wu, Xiwei Fan, Ross Crawford, Yin Xiao, Indira Prasadam

**Affiliations:** ^1^Centre for Biomedical Technologies, Faculty of Engineering, Queensland University of Technology, Brisbane, Queensland, Australia.; ^2^Department of Orthopaedic Surgery, the Second Xiangya Hospital, Central South University, Changsha, Hunan, China.; ^3^The Prince Charles Hospital, Orthopedic Department, Brisbane, Queensland, Australia.; ^4^Australia-China Centre for Tissue Engineering and Regenerative Medicine, Queensland University of Technology, Brisbane, Queensland, Australia.

**Keywords:** energy metabolism, osteoarthritis, articular cartilage, chondrocyte, glycolysis, oxidative phosphorylation

## Abstract

Articular cartilage function depends on the temporal and zonal distribution of coordinated metabolic regulation in chondrocytes. Emerging evidence shows the importance of cellular metabolism in the molecular control of the cartilage and its dysregulation in degenerative diseases like osteoarthritis (OA). Compared to most other tissues, chondrocytes are sparsely located in the extracellular matrix, lacking the typical proximity of neural, vascular, and lymphatic tissue. Making up under 5% of the total tissue weight of cartilage, chondrocytes have a relative deficiency of access to nutrients and oxygen, as well as limited pathways for metabolite removal. This makes cartilage a unique tissue with hypocellularity, prolonged metabolic rate, and tissue turnover. Studies in the past decade have shown that several pathways of central carbon metabolism are essential for cartilage homeostasis. Here, we summarised the literature findings on the role of cellular metabolism in determining the chondrocyte function and how this metabolic dysregulation led to cartilage aging in OA and provided an outlook on how the field may evolve in the coming years. Although the various energy metabolism pathways are inextricably linked with one another, for the purpose of this review, we initially endeavoured to examine them individually and in relative isolation. Subsequently, we comment on what is known regarding the integration and linked signalling pathways between these systems and the therapeutic opportunities for targeting OA metabolism.

## 1. Introduction

Osteoarthritis (OA) is a degenerative joint disease with pathological changes in all joint compartments: cartilage, subchondral bone, and synovium [1]. With no disease-modifying medications available at the moment and an increasing prevalence of OA, risk factors such as obesity and ageing will continue to bear a more significant burden in the future. As a result, it is critical to develop treatment targets, techniques, and/or medicines capable of effectively reversing or halting the disease process. The cause of OA is still unclear, with many pathways and molecules interplaying between mechanical, genetic, metabolic factors, and inflammatory mechanisms. Emerging evidence indicates that cellular metabolism dysfunction is involved in many chronic diseases, such as diabetes, cancer, and Alzheimer's disease [2-4]. Often the metabolism changes are a result of a failure of energy production, biomolecule synthesis (amino acids, nucleotides, fatty acids, lipids), mitochondria and reactive oxygen species (ROS) regulation, or dysregulation of energy-sensing signalling pathways such as mammalian target of rapamycin (mTOR), peroxisome proliferator-activated receptor gamma (PPAR-γ), nicotinamide adenine dinucleotide (NAD^+^) and AMP-activated protein kinase (AMPK). Series of these altered molecular events ultimately affect the cellular phenotype by altering adenosine triphosphate (ATP) production, the primary energy currency in living cells, and the critical factor supporting energy-consuming processes such as growth, catabolic, and differentiation related functions of a tissue.

In comparison to the synovial tissue surrounding the joint and the underlying subchondral bone, articular cartilage is a hypocellular, avascular, aneural, and alymphatic tissue with decreased access to oxygen and glucose. Chondrocytes, the only cell type present in the articular cartilage, are the primary controller of cartilage tissue metabolism. Healthy cartilage needs to maintain a delicate energy balance between anabolic and catabolic activities, which is essential for long-term tissue integrity and to maintain its ability to repair itself and to resist extracellular matrix (ECM) degeneration. Chondrocyte metabolic alterations are involved in various OA phenotypes, including aging, obesity, and trauma-related OA, and may be essential for disease development [1]. The enzymes and signalling pathways in chondrocytes, particularly central carbon metabolism, determine cellular bioenergetics and have a substantial impact on a series of cellular functions, including proliferation, ECM production, apoptosis, autophagy, and inflammation [5-10]. However, the fundamental question of how chondrocytes maintain energy metabolism is challenging to elucidate as the energy-producing biochemical pathways overlap and quickly shift depending on their microenvironment.

Energy metabolism produces energy from nutrients needed to maintain essential cellular homeostasis, precise cellular activity, and normal function. Metabolism encompasses a series of interrelated pathways that play a role in the presence or absence of oxygen, including glycolysis, tricarboxylic acid (TCA) cycle, pentose phosphate pathway, fatty acid oxidation, fatty acid synthesis, and amino acid metabolism. Different pathways involved in metabolism are universal to most cells and engage in crosstalk by sharing the same substrates and enzymes [11]. For example, glucose and fatty acids compete for acetyl-CoA as a source of oxidative energy in the TCA cycle and consequent mitochondrial oxidative energy generation, but increasing fatty acid availability significantly inhibits glucose oxidation. In addition to producing ATP, energy metabolism is also vital for promoting inflammation and cellular senescence through several enzymes and signalling pathways. This includes acting as transcription factor ligands, such as peroxisome proliferator-activated receptor α (PPARα) and serving as precursors for fatty acid derivatives, as well as affecting insulin resistance and apoptosis. Therefore, it is now widely accepted that metabolic therapies can be applied against a vast array of diseases. This article will address four main issues: (1) During the development of OA, what is the actual bioenergetic balance of cartilage cells between glycolysis and oxidative phosphorylation (OXPHOS)? (2) What are factors controlling the balance between glycolysis and OXPHOS? (3) What signalling mechanisms are responsible for metabolic changes in cartilage? (4) Novel tools for future energy metabolism investigations are reviewed, and future directions for OA metabolism research are discussed.

## 2. What is the bioenergetic balance between glycolysis and OXPHOS in normal and OA chondrocytes?

### 2.1. Glycolytic capacity of chondrocytes

**Figure 1.**
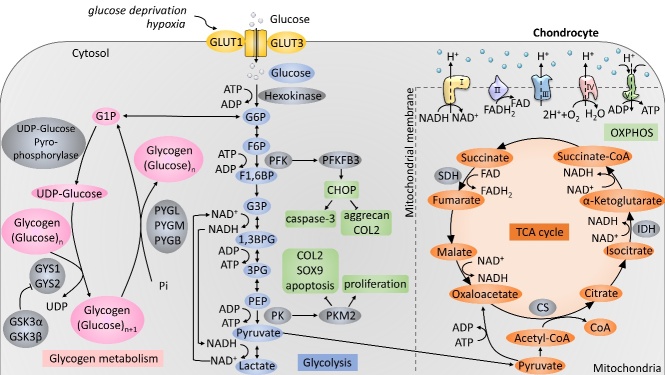
**Schematic illustration of central carbon metabolism in chondrocytes**. Glucose enters the cell through transporters like glucose transporter 1 (GLUT1) and glucose transporter 3 (GLUT3) and can be converted to glyceraldehyde 6-phosphate (G6P) by hexokinase. G6P derived pyruvate via glycolysis, thereby producing lactate or entering the tricarboxylic acid (TCA) cycle and providing energy for the cells. On the other hand, G6P acts as a starter for glycogen metabolism to support glycogen synthesis and phosphorylation

Cartilage energy metabolism relies on two primary substrates: glucose and oxygen. Glucose is the primary metabolic fuel and structural precursor in cartilage because more than 75% of total cellular ATP is derived from the glycolytic pathway, while the rest is produced by OXPHOS (Fig. 1) [1]. Cartilage expresses all the enzymes needed for glucose metabolism. In chondrocytes, glucose is transported into the cell with the help of the glucose transporter (GLUT), allowing passive diffusion across the cell membrane. The most highly expressed GLUT families in chondrocytes are GLUT1 and GLUT3. GLUT1 is upregulated in response to hypoxia and glucose deprivation but decreases in high glucose environments, increasing the ability to uptake glucose under low oxygen conditions and balancing glucose levels in cells [12]. Expression of GLUT3 is not changed by either anabolic or catabolic stimuli [13]. It is still unclear how levels of GLUT1 are altered in OA, as current research reports conflicting findings. 2-Deoxy-d-glucose uptake increases more intensely in normal chondrocytes than in OA chondrocytes, indicating that glucose transport is decreased in OA chondrocytes [14]. However, another study reported that glucose transport capacity was not affected in normal or OA chondrocytes, while another team indicated that GLUT1 was increased and was associated with increased cartilage degradation [15,16]. GLUT1 is essential for embryo growth, cartilage formation, and skeletal system development [17]. Chondrocytes can regulate GLUT1 expression in response to changes in extracellular glucose concentration on the cell membrane, hence preserving proper cartilage formation. [18]. Genetic deletion of GLUT1 can lead to achondroplasia, and continuously increased expression of GLUT1 can degrade cartilage by increasing glucose uptake resulting in the production of excessive advanced glycation end products leading to proteoglycan depletion, which is a primary structural component of the ECM [18].

Hexokinase, phosphofructokinase (PFK), and pyruvate kinase (PK) are rate-limiting enzymes. Hexokinase is a glucose sensor, the first step of glycolysis that converts glucose into glucose 6-phosphate (G6P) [19]. Hexokinase II is increased in OA chondrocytes by activating transforming growth factor beta 1 (TGF-β1) [20]. PFK catalyses the important glycolytic "committed" step, which converts fructose 6-phosphate (F6P) and ATP into fructose 1,6-diphosphate (F16BP) and adenosine diphosphate (ADP), maintaining the convenience of transporting the downhill concentration gradient of glucose into the cell. PFK can regulate glycolysis through allosteric inhibition so that cells can alter their glycolysis rate in response to their energy requirements. Expression of 6-phosphofructose-2-kinase/fructose-2,6-bisphos-phatase 3 (PFKFB3) in human OA cartilage tissue was downregulated [21]. In addition, PFKFB3 improves the cell viability of OA cartilage explants and chondrocytes through the phosphoinositide 3-kinase (PI3K)/protein kinase B (AKT)/C/EBP homologous protein (CHOP) signalling pathway, reduces the activation of caspase 3, and promotes expression of aggrecan and type II collagen (COL2) [21]. PK is the enzyme involved in the last step of glycolysis. It catalyses the final rate-limiting step of glycolysis, which transfers phosphoenolpyruvate (PEP) to ADP, producing one molecule of pyruvate and one molecule of ATP. A previous study showed that pyruvate kinase M2 (PKM2) in OA cartilage chondrocytes was upregulated, while ATP production was reduced. PKM2 inhibition prevents the proliferation of OA chondrocytes, promotes cell apoptosis, and lowers expression levels of COL2α1 and sex-determining region Y-type box transcription factor 9 (SOX9), indicating its potential role in the pathogenesis of OA [10]. When PKM2 is overexpressed in OA cartilage chondrocytes, lactate dehydrogenase transforms the pyruvate molecule to lactate in the cytoplasm, generating two ATP molecules. This results in lactate build-up and the creation of an acidic microenvironment [10]. The acidic micro-environment (overall average cartilage pH=6.60) has been shown to inhibit matrix synthesis in chondrocytes (pH<7.1) and may promote cartilage degeneration in OA [22]. Upregulation of lactate production was also found to induce inflammation in chondrocytes by activating the nuclear factor kappa B (NF-κB) signalling pathway, which can further lead to reprogramming of cell metabolism and OA [8].

The changes that occur in different substrates and the overall alterations of bioenergetics profile in OA cartilage are still unclear. Wang et al. first reported that basal respiration and maximal respiration were decreased in OA chondrocytes, while Michelle et al. further found that respiration rate gradually reduced along with the progress of cartilage damage [23,24]. Similar results were also found in IL-1β-treated chondrocytes, in which basal glycolysis levels were increased compared to that in untreated chondrocytes [8,25]. IL-1β treatment causes metabolic reprogramming, mimicking the Warburg effect, and was expanded according to some previous reports on chondrocyte metabolism [10]. These findings indicate an increased glycolytic rate in OA chondrocytes. However, the levels of IL-1β used in vitro are 10-fold what is present in OA in vivo, and currently there is no report focusing on bioenergetics profile in OA animal model. The role of energy metabolism may be different in OA animal model and OA patients. A summary of all related bioenergetics profiles available on chondrocytes was shown below inTable 1.

**Table 1:** Altered chondrocytes bioenergetics profile in OA context.

Species	Treatment	Results		Ref.
		ECAR	OCR	
**Mice**	IL-1β *in vitro*	Glycolysis increased	Basal respiration and maximal respiration decreased	[8]
**Mice**	Sirt5^-/-^ *in vivo*	Basal glycolysis and compensatory glycolysis decreased	Basal respiration, maximal respiration and ATP production decreased	[5]
**Mice**	Paraquat *in vitro*	N/A	Basal respiration and maximal respiration decreased	[10]
**Mice**	Sod2^-/-^ *in vivo*	N/A	Basal respiration and maximal respiration decreased	[10]
**Human**	Doxycycline *in vitro*	N/A	Low dose of doxycycline increased maximal respiration and spare capacity while high dose of doxycycline reduced ATP production	[26]
**Mice**	Doxycycline *in vitro*	N/A	Basal respiration and maximal respiration decreased	[27]
**Bovine**	IL-1β *in vitro*	N/A	Basal respiration and maximal respiration decreased	[25]
**Human**	Comparing normal VS OA human chondrocytes *in vitro*	N/A	Oxygen consumption decreased in OA chondrocytes	[23]
**Human**	BMP2 *in vitro*	N/A	Basal respiration and maximal respiration increased	[20]
**Mice**	Twinkle transgenic *in vivo*	N/A	Basal respiration, maximal respiration, ATP production and spare capacity decreased	[28]
**Bovine**	Comparing minimal to severe damage cartilage *in vitro*	N/A	Basal respiration and maximal respiration decreased in severe damage cartilage	[24]

Abbreviations: Sod2: Superoxide dismutase 2; Sirt5: Sirtuin 5; BMP2: Bone morphogenetic protein 2; IL-1β: Interleukin-1β; OA: Osteoarthritis; ECAR: Extracellular acidification rate; OCR: Oxygen consumption rate; ATP: Adenosine triphosphate.

### 2.2. Glycogen metabolism during OA development.

**Figure 2.**
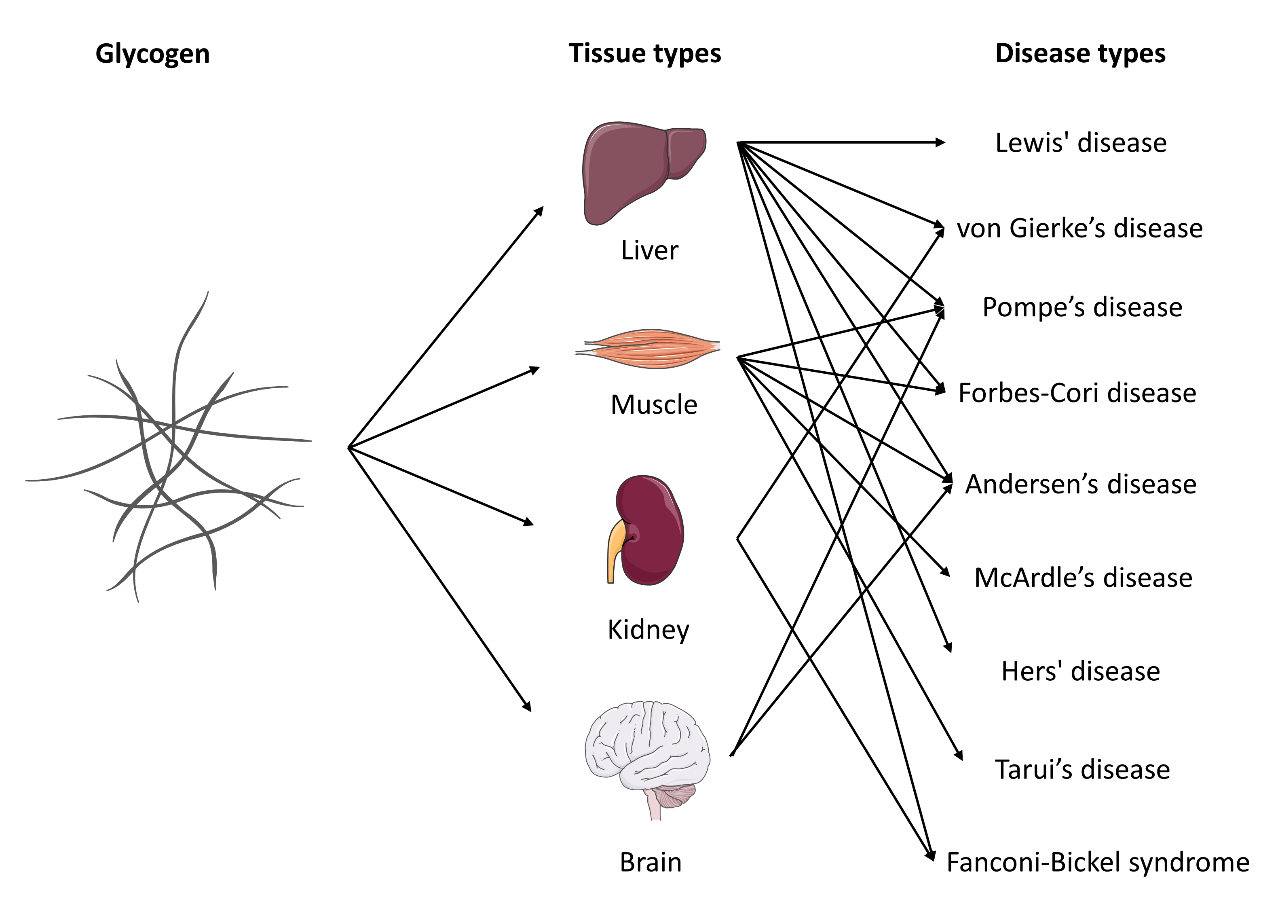
**Summary of glycogen storage diseases**. Glycogen is a multibranched polysaccharide of glucose that serves as energy storage in specific tissue types such as the liver, muscle, kidney, and brain. Improper form or release of glycogen in different tissues causes a group of inherited genetic disorders in the body.

Glycogen is a highly branched polymer of glucose that is utilized to store and release energy efficiently. In humans, glycogen is primarily produced and stored in the cells of the liver and skeletal muscle. The breakdown of liver glycogen increases the amount of blood sugar available to other tissues, while the phosphorylation of muscle glycogen rapidly increased the glucose level available to produce enough energy for muscle movement. Cartilage is a soft tissue without any blood vessels; therefore, blood glucose can hardly facilitate glucose metabolism in cartilage. However, to produce a massive extracellular matrix with less than 5% weight of cells, chondrocytes require plenty of energy. The activity of glycogen phosphorylation in growth plates has been established but no report in articular cartilage tissue [29]. However, the critical aspects of glycogen metabolism in articular cartilage are still unclear from the available literature. Lee et al. found that in an anoxic environment, the ratio between glucose uptake and lactate production could be more than 1:10, indicating that another energy source is used to produce more lactate and energy without glucose uptake in cartilage such as glycogen [30]. Dysfunction of glycogen metabolism in different tissues may cause different glycogen storage diseases, such as Lewis' disease in the liver, von Gierke’s disease in the kidney, McArdle's disease in the muscles, and Pompe’s disease in the nervous system (Fig. 2). Future studies should focus on whether glycogen exists in articular cartilage and the role of glycogen in chondrocyte bioenergetic and cellular functions.

Controlling glycogen synthase via glycogen synthase kinase 3 (GSK3) is a critical regulatory step in glycogen production (Fig. 1). GSK has two isoforms, GSK3α and GSK3β. GSK3 activity is chondroprotective in OA. Normal cartilage was negative for phosphorylated-GSKβ staining in all layers, while most phosphorylated-GSKβ-positive cells in OA cartilage were in the middle and deep layers. In addition, compared to chondrocytes in nonobese OA patients, chondrocytes in obese OA patients exhibited higher levels of phosphorylated GSKβ [31]. GSK3α and GSK3β can be inactivated by a reversible phosphorylation reaction. GSK3β deletion causes upregulation of GSK3α *in vivo*[32]. In addition, GSK3 affects glycogenesis and influences ROS production, oxidative damage, cell proliferation, DNA damage response, chondrocyte senescence, and hypertrophy [31]. Differential expression of glycogen synthesis enzymes provides evidence for altered glycogen synthesis in OA chondrocytes, and the effect of GSK3 on chondrocyte function indicates a link between glycogen and OA progression.

### 2.3. Tricarboxylic acid cycle and OXPHOS

Up to 25% of total chondrocyte ATP is derived from oxidative phosphorylation (Fig. 1) [1]. It was reported that malate, citrate, 2-oxoglutarate, succinate, and fumarate were reduced in mouse cartilage in response to increasing age [33]. As OA is an age-related degenerative disease, these results might correspond with cartilage degeneration. The pro-inflammatory cytokine IL-1β is one of the most common factors used to induce OA in chondrocytes *in vitro*. Haseeb et al. found that IL-1β significantly suppressed the activity and expression of isocitrate dehydrogenases 1 and 2 (IDH1 and IDH2, respectively) in primary human chondrocytes, which correlated with reduced α-ketoglutarate levels. IDH activates the first oxidative step within the TCA cycle and is one of the key rate-limiting enzymes in the TCA cycle. In contrast to the above study, IDH2 and citrate synthase were significantly increased in Kashin-Beck disease (an endemic degenerative osteoarthritis) chondrocytes compared to normal cells [34]. These differences could be related to different disease pathologies themselves.

Mitochondrial dysfunction and oxidative stress are characteristics of OA. Compared to chondrocytes from healthy cartilage, mitochondrial DNA (mtDNA) damage is increased in OA chondrocytes, while mtDNA repair capacity and cell viability are reduced, and apoptosis is increased [35]. Pro-inflammatory cytokines (IL-1β and TNF-α) destroy mitochondrial functions by causing DNA damage in the mitochondria, resulting in decreased energy production and DNA transcription [36]. Changes in mitochondrial membrane potential are detected in chondrocytes of OA patients. Compared to chondrocytes of healthy individuals, OA chondrocytes exhibit decreased complexes II and III and decreased mitochondrial membrane potential [37]. Although the majority of ATP in OA chondrocytes is generated via glycolysis rather than oxidative phosphorylation, mitochondrial ROS contributes to the cell's redox equilibrium, which is conducive to glycolysis [38]. Mitochondrial uncoupling has been demonstrated to decrease lactate generation in hypertrophic chondrocytes, implying that glycolysis is dependent on mitochondria to function properly [39].

**Figure 3.**
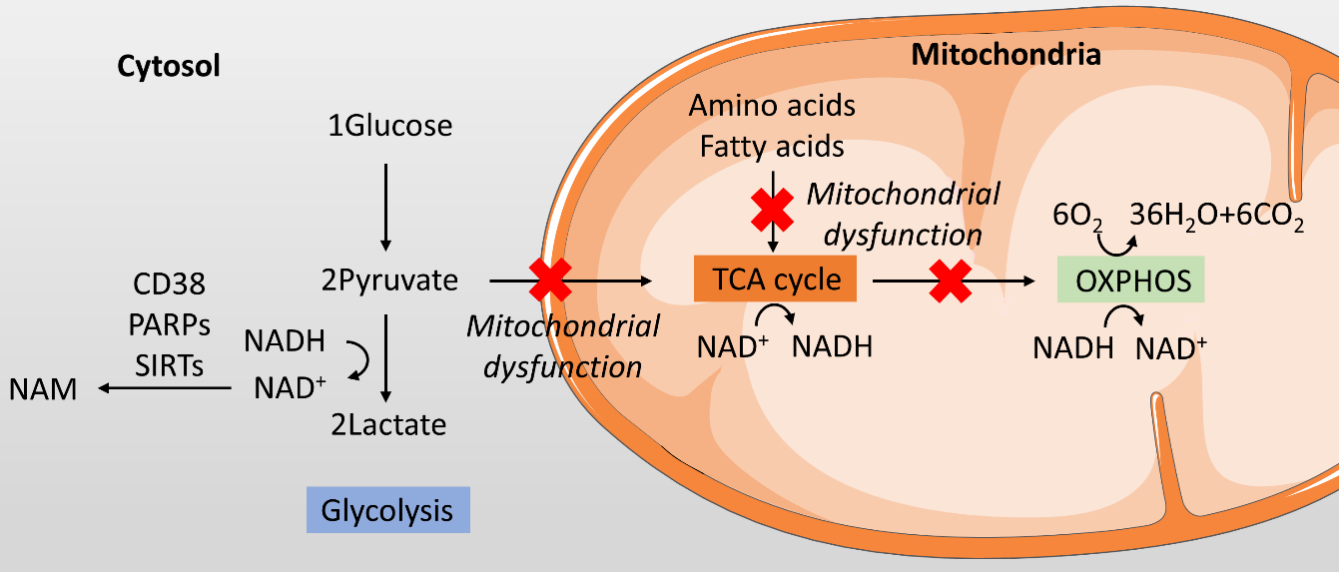
**The balance between glycolysis and oxidative phosphorylation in chondrocytes**. In chondrocytes, whether glucose entering glycolysis or oxidative phosphorylation is affected by various factors, including oxygen level, the nicotinamide adenine dinucleotide (NAD^+^, oxidized form)/NADH (NAD^+^reduced form) ratio, and mitochondrial function.

## 3. What factors control the balance between glycolysis and OXPHOS?

The uncoupling of glycolysis and OXPHOS occurs through the partitioning of pyruvate and lactate [40]. Whether pyruvate converting into lactate or entering the TCA cycle controls the balance between glycolysis and OXPHOS. Three main factors may contribute to this balance: oxygen level, the nicotinamide adenine dinucleotide (NAD^+^, oxidized form)/NADH (NAD^+^reduced form) ratio, and mitochondrial function (Fig. 3).

First, oxygen is not needed in the glycolysis pathway, which converts one molecule of glucose into two molecules of lactate. In OXPHOS, six molecules of oxygen are needed to catabolize one molecule of glucose completely. When cells are in a hypoxic environment, pyruvate is converted into glycolysis in the cytosol instead of entering the TCA cycle in the mitochondria [41]. However, cartilage is a relatively hypoxic tissue. Oxygen levels vary from 6% on the surface to 1% in the deep zone [1]. It is believed that the Warburg effect exists in chondrocytes, and even in the presence of oxygen, the rate of glucose uptake and the preferential production of lactate are also increased [8,10]. The glycolysis rate increases when chondrocytes are treated with IL-1β as determined using the Seahorse technique, where oxygen is sufficient inside the Seahorse Extracellular Flux Analyser [8]. Therefore, the oxygen level is not the only factor controlling the balance between glycolysis and OXPHOS. There are likely other factors, especially in chondrocytes.

NAD^+^/NADH ratio is also one of the main factors affecting this balance. When pyruvate converts into lactate, NADH loses H^+^ and produces NAD^+^ in the cytosol. In an oxygen-sufficient environment, NADH, H^+^, and oxygen can enter OXPHOS and produce ATP and H_2_O [42]. In the hypoxic environment, NADH and H^+^ cannot be oxidized in the mitochondria. Pyruvate is therefore fully oxidized, and no lactate is produced. Pyruvate then produces lactate instead of combining with NADH. In addition, NAD^+^ levels also induce a direct negative regulatory effect between pyruvate and lactate [43]. High levels of NAD^+^ can directly inhibit pyruvate transfer into lactate, while low NAD^+^ levels increase lactate production. NAD^+^ levels are decreased in degenerative diseases, which may be due to the increase in NAD^+^ consumption enzymes [44]. One of the main NAD^+^ consumption enzymes, P1), was reduced by incubating chondrocytes with IL-1β, indicating a decrease in NAD^+^ in OA [45]. Together, the low level of NAD^+^ and high level of NADH continuously push pyruvate to convert into lactate and increase glycolysis in chondrocytes, especially under OA conditions.

Third, mitochondrial dysfunction also controls the balance between glycolysis and OXPHOS, not only in utilizing glucose but also in amino acids and fatty acids. The catabolism of glucose, amino acids, and fatty acids shares the same pathway with the TCA cycle and OXPHOS through acetyl-CoA, while amino acids can also be broken down into substrates in the TCA cycle, such as oxaloacetate, fumarate, succinyl-CoA, and α-ketoglutarate [46,47]. The significance of mitochondrial dysfunction in the development of OA has attracted widespread attention [9]. Many studies have shown that mitochondrial function in OA chondrocytes is impaired, leading to increased chondrocyte apoptosis and decreased secretion of type II collagen [48,49]. Mitochondrial dysfunction directly affects or even shuts down the normal function of the TCA cycle and OXPHOS, causing concomitant metabolic reprogramming of the TCA cycle. Mitochondrial membrane potential is reduced in response to mitochondrial dysfunction, which further inhibits pyruvate crossing the membrane [50]. Pyruvate can, therefore, only convert into lactate and flow through glycolysis in OA.

**Figure 4.**
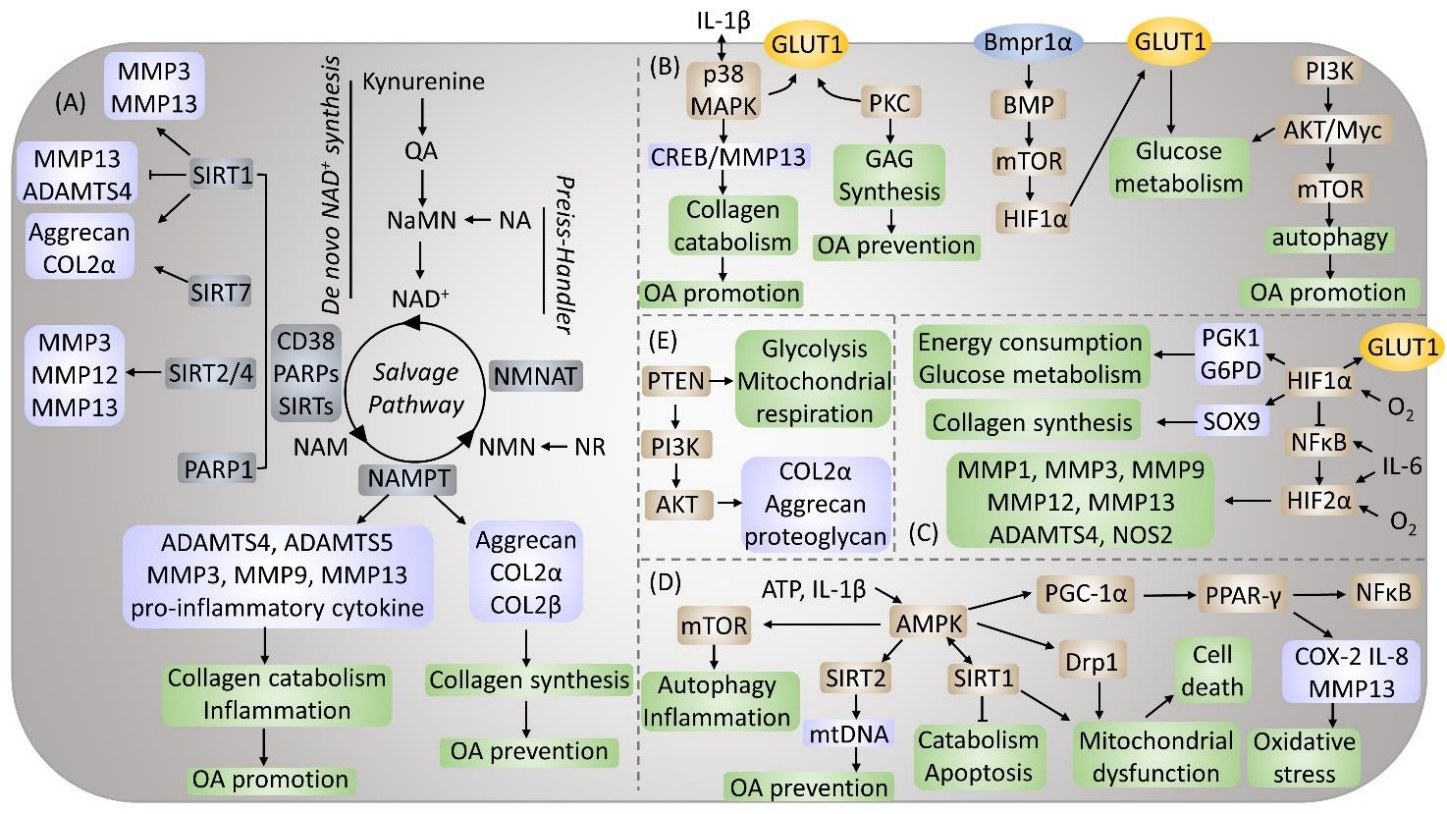
**Molecular interface between bioenergetics, signaling pathways, and chondrocytes metabolism**. Regulation of chondrocytes metabolism by (A) Nicotinamide adenine dinucleotide metabolism pathway, (B) Mitogen-activated protein kinase (MAPK) and mechanistic target of rapamycin (mTOR) signaling pathways, (C) Hypoxia-inducible factors (HIFs) pathway, (D) The AMP-activated protein kinase (AMPK) signaling pathway and (E) Phosphatase and tensin homologue (PTEN)/ Phosphoinositide 3-kinase (PI3K)/ Protein kinase B (AKT) signaling pathway.

## 4. The molecular interface among bioenergetics, signaling pathways, and chondrocyte metabolism

### 4.1. The nicotinamide adenine dinucleotide metabolism pathway in OA

NAD^+^ is a coenzyme of redox reactions, making it the centre of energy metabolism [51]. The ability of NAD^+^ to accept hydrogen ions to form its reduced form NADH is essential for the metabolic reactions of all life forms. It is involved in regulating dehydrogenases that are engaged in a variety of catabolic processes, including glycolysis, the TCA cycle, and fatty acid oxidation [51]. NAD^+^ concentrations depend on the cellular redox state (NAD^+^/NADH ratio), the rates of NAD^+^ synthesis, and NAD^+^ consumption (Fig. 4A). NAD^+^ levels decrease during aging and senescence, which might be due to the deficiency of NAD^+^ synthesis or the increase in the NAD^+^ consumption rate [52]. Although NAD^+^ has been widely studied in age-related diseases, how NAD^+^ is altered in OA is still unclear. As the most common degenerative disease, more research is needed to promote our understanding of the role of NAD^+^ in OA.

NAD+ can be generated de novo from tryptophan via the kynurenine pathway or the Preiss-Handler pathway from vitamin precursors such as nicotinic acid (NA) [53]. The majority of cells lack all of the enzymes necessary to convert tryptophan to NAD^+^ via the kynurenine pathway. The majority of tryptophan is metabolized in the liver to nicotinamide (NAM), which is then released into the serum and absorbed by peripheral cells via the NAD^+^ salvage pathway [54]. Nicotinamide phosphoribosyl-transferase (NAMPT) is the rate-limiting enzyme in the NAD^+^ salvage pathway. Yang et al. found that both human and mouse OA chondrocytes produced NAMPT, and IL-1β treatment increased the synthesis of NAMPT [55,56]. NAMPT triggered excessive release of A Disintegrin and Metalloproteinase with Thrombospondin motifs (ADAMTS)-4 and ADAMTS-5 expression and matrix metalloproteinase (MMP) 3, MMP9, and MMP13 synthesis. FK866 and APO866 are two main NAMPT inhibitors. Evans et al. established that NAMPT is a critical regulator of inflammation, cartilage catabolism, and bone erosion and stressed the therapeutic potential of FK866 in inflammatory arthritis [57]. The effect of NAMPT in OA is still unclear, as current research reports conflicting findings. In animal studies, Yang and Busso et al. utilized FK866 to inhibit NAMPT, resulting in reduced MMP3, MMP9, and MMP13 production and decreased pro-inflammatory cytokine release [55,58]. However, another study by Mona et al. reported that inhibition of NAMPT reduces cartilage-specific gene expression, such as Aggrecan, COL2α, and COL2β [59].

NAD^+^ is continually turned over by three classes of NAD^+^-consuming enzymes: (i) sirtuins (SIRTs), (ii) ADP-ribose transferases (ARTs) and PARPs, and (iii) cyclic ADP-ribose (cADPR) synthases (CD38 and CD157) [60]. The mammalian SIRT family consists of 7 members, SIRT1-7, which have distinct subcellular localization patterns and enzymatic activities [61]. SIRT regulates gene expression through direct deacetylation of histones and transcription factors to regulate chromatin function [62]. SIRT1, 2, 4, and 7 were essential regulators of cartilage homeostasis and OA development. SIRT1 is reported to increase NAMPT expression, while the enzymatic activity of NAMPT stimulates the formation of NAD^+^, a critical cofactor for SIRT1 deacetylase [63,64]. Thus, a SIRT1-NAMPT-NAD^+^-SIRT1 positive feedback loop signalling pathway was formed and may contribute to OA progression. In addition, SIRT1 also directly mediates MMP3 and MMP13 production in chondrocytes, resulting in OA promotion [65]. However, Nishida et al. reported the opposite result using the SIRT1 activator SRT1720 in experimental OA mice. They found that SIRT1 attenuated the progression of OA by decreasing MMP13 and ADAMTS5 and increasing COL2α1 and Aggrecan in chondrocytes [66]. Hypoxia-inducible factor (HIF)-2α is an important catabolism regulator of OA cartilage destruction, which acts by upregulating the expression of matrix-degrading enzymes in chondrocytes [67]. SIRT2 and SIRT4 expression levels in chondrocytes are positively linked with the stability and transcriptional activity of HIF-2α. This mutual regulation, the HIF-2α-NAMPT-NAD^+^-SIRT axis, is necessary for the expression of MMP3, MMP12, and MMP13 and the destruction of OA cartilage [67]. On the other hand, it has recently been discovered that SIRT7 exerts a chondroprotective effect by increasing the formation of glycosaminoglycan-rich extracellular matrix and the production of extracellular matrix components, such as COL2α1 and aggrecan [68]. Among all PARPs (17 proteins), the function of PARP4-17 in NAD^+^ homeostasis and global metabolism in cells has not been thoroughly investigated, and they are thought to be less critical in modulating intracellular NAD^+^ levels [51]. PARP1-3 localizes to the nucleus in response to similar NAD^+^ levels and DNA damage by sharing a similar catalytic domain [69]. PARP1 was reduced by incubating chondrocytes with IL-1β, which could be reversed by a caspase inhibitor, suggesting that this process is caspase-dependent [70]. However, this is still controversial, as Sun et al. demonstrated that IL-1β stimulates chondrocytes and leads to a significant upregulation of PARP1, while PARP1 inhibition prevents IL-1β-induced inflammation by inhibiting the IL-1R/NF-кB signalling pathway and reducing expression of MMPs in human articular chondrocytes [45]. Meanwhile, PARPs and SIRTs compete for the total NAD^+^ pool. In a mouse model, mice treated with PARP inhibitors or PARP1 and PARP2 knockout mice exhibited increased SITR1 activity and NAD^+^ levels [71]. There is currently little known about CD38 or CD157 in chondrocytes during the process of OA. CD38 is thought to mediate immunity; however, it is unclear whether the function of the CD38 enzyme is necessary for its function or to what extent these functions depend on NAD^+^[51]. The overview of metabolic changes in OA was summarized inTable 2.

### 4.2. Aerobic glycolysis in chondrocytes: MAPK and mTOR signalling pathways

It is still unclear how aerobic glycolysis is altered in OA chondrocytes; however, upregulation of GLUT1 was observed when Alexander et al. treated chondrocytes with IL-1β, which enhanced chondrocyte uptake of glucose and increased glycolysis [80]. Signal transduction analysis demonstrated that IL-1β stimulates glucose transport via activation of p38 MAPK and protein kinase C (PKC). p38 MAPK is activated by environmental stress and inflammatory cytokines, including IL-1β. In addition, previous studies reported that activation of the p38/MAPK signalling pathway might lead to the expression of pro-inflammatory cytokines, which promotes activation of p38 and plays a key role in the development of OA [73]. At the same time, elevated p38 can also activate the p38/cyclic adenosine monophosphate (cAMP) response element binding protein (CREB)/ MMP13 axis in OA chondrocytes and may cause cartilage degeneration [81]. In chondrocytes, inhibiting p38 MAPK decreases downstream inflammatory cytokine production and MMP expression [82]. Previous reports that PKC activation influences glycosaminoglycan production in chondrocytes offered indirect support for the participation of PKC in glucose transport regulation [74]. However, individual PKC isoforms may have different functions, and there is no mention of which specific PKC isoforms are relevant [83]. In addition, deletion of GLUT1 severely impaired chondrocyte proliferation and hypertrophy via the bone morphogenetic protein (BMP)/mTOR/HIF-1α signalling pathway [84]. GLUT1 was also found to be a downstream effector of BMP signalling in cartilage development because the loss of BMP receptor 1α (Bmpr1α) in chondrocytes significantly reduced levels of GLUT1 in the body, while recombinant BMP2 increased levels of GLUT1 mRNA and protein. Thus, blocking GLUT1 or other glycolytic pathway components from causing aberrant hypertrophy may benefit in preventing the progression of OA. According to existing information, the PI3K/AKT/mTOR signalling pathway is required for normal joint tissue metabolism and plays a role in the OA development [85]. By activating Myc and AKT and inactivating p53, the upregulation of glycolytic enzymes and glucose transporters is believed to cause enhanced glycolysis [75]. mTOR is a serine/threonine-protein kinase distributed downstream of AKT. It has been demonstrated that activating mTOR inhibits autophagy and that the inactive mTOR pathway induces autophagy [86]. Inhibition of the PI3K/AKT/mTOR signalling pathway promotes autophagy in articular chondrocytes and dampens the inflammatory response in OA (Fig. 4B) [87].

**Table 2:** Overview of metabolic changes in OA chondrocytes.

Metabolic Signaling Pathway	Alteration in OA	Relationship with Metabolism	Ref.
**Nicotinamide adenine dinucleotide pathway**			
**NAMPT**	Increase	Produce NAD^+^	[55]
**SIRT1**	Decrease	Consume NAD^+^	[72]
**PARP1**	Controversial	Consume NAD^+^	[45,70]
**MAPK and mTOR pathway**			
**p38**	Increase	Upregulate GLUT1, activate p38/cAMP/CREB/MMP13 axis	[73]
**PKC**	Increase	Upregulate GLUT1, regulate glycosaminoglycan synthesis	[74]
**AKT**	Increase	Upregulate glycolytic enzymes and glucose transporters	[75]
**HIFs pathway**			
**HIF-1α**	Increase	Increase glycolysis and reduce mitochondrial respiration	[76,77]
**AMPK pathway**			
**AMPK**	Decrease	Decrease ATP production and increase ATP consumption	[78]
**PTEN/PI3K/AKT pathway**			
**PTEN**	Increase	Reduce proteoglycan synthesis, glycolysis and mitochondrial respiration	[79]

Abbreviations: OA: Osteoarthritis; NAMPT: Nicotinamide phosphoribosyltransferase; SIRT1: Sirtuin 1; PARP1: Poly(ADP-ribose) polymerase 1; PKC: Protein kinase C; AKT: Protein kinase B; HIF-1α: Hypoxia-inducible factor 1α; AMPK: AMP-activated protein kinase; mTOR: Mammalian target of rapamycin; ATP: Adenosine triphosphate; NAD^+^: Nicotinamide adenine dinucleotide; GLUT1: Glucose transporter 1; cAMP: Cyclic adenosine monophosphate; CREB: Response element binding protein; MMP13: Matrix metalloproteinase 13.

### 4.3. HIFs and glycolysis in chondrocytes

HIFs are the primary regulators of the adaptive response to hypoxia, regulating oxygen homeostasis and metabolic activation of genes through transcriptional activation [88]. HIF heterodimers consist of HIFα (isoforms HIF-1α, HIF-2α, HIF-3α) and HIFβ (also termed aryl hydrocarbon receptor nuclear translocator, ARNT) subunits [89]. During cartilage development and regeneration, articular chondrocytes are located in an avascular, hypoxic, and dystrophic milieu and respond to oxygen changes via the transcription factor HIF-1α [90]. HIF-1α is a critical modulator of chondrocyte oxygen homoeostasis and responds to changes in oxygen supply during cartilage growth or injury [91]. Under normal oxygen conditions, HIF-1α is continuously generated and depleted and has a relatively short half-life (6 minutes). Under conditions of hypoxia or reduced oxygen concentration, the degradation rate of HIF-1α decreases, prolyl hydroxylation is inhibited, and proteasome degradation of HIF-1α is prevented. Thus, HIF-1α accumulates and is carried into the nucleus under hypoxia, activating target genes that enhance glucose absorption and lactate generation while decreasing mitochondrial respiration [90]. Compared to the entire area, in OA cartilage, expression of HIF-1α is higher in the degenerated area [76]. Chondrocytes produce the most ATP through anaerobic glycolysis [30]. HIF-1α plays a crucial role in ATP synthesis as it regulates the expression of at least 13 genes involved in anaerobic glycolysis [92]. HIF-1α modulates a variety of enzymes involved in anaerobic glycolysis in chondrocytes, including GLUT1, phosphoglycerate kinase 1 (PGK1), and glucose-6-phosphate dehydrogenase (G6PD) [77]. G6PD is the rate-limiting enzyme in the pentose-phosphate pathway, regulating energy expenditure and glucose metabolism [93]. HIF-1α also activates ECM synthesis genes, such as SOX9 [77]. Recent studies have also shown that by inhibiting NF-κB signalling, HIF-1α has an anti-degradation function in maintaining articular cartilage [94]. HIF-1α can also upregulate microsomal prost-aglandin E synthase 1 (mPGES-1), indicating the potential role of HIF-1α in the metabolism of OA chondrocytes [95].

HIF-2α, encoded by the endothelial PAS domain protein 1 (EPAS1), is considered harmful to articular cartilage [96]. Compared to non-diseased cartilage in mice and humans, HIF-2α expression in OA cartilage is higher [97]. IL-1β induces c-Jun N-terminal kinase (JNK) phosphorylation, representing a key pathway for HIF-2α expression [98]. It has also been found that IL-6 acts as an important mediator of HIF-2α-induced OA cartilage breakdown in mice by regulating levels of MMP3 and MMP13 [99]. HIF-2α regulates the expression of key catabolic genes, including MMP1, MMP3, MMP9, MMP12, MMP13, ADAMTS4, and NOS2, playing a role in OA progression [100]. Expression of HIF-2α is regulated by the NF-κB signalling pathway while inhibiting NF-κB activation reduces expression of HIF-2α and alleviates osteoarthritic cartilage destruction (Fig. 4C) [101].

### 4.4. The AMP-activated protein kinase (AMPK) signalling pathway in chondrocyte metabolism and mitochondrial dysfunction

AMPK is a sophisticated sensor of low cellular ATP levels in eukaryotes that phosphorylates particular enzymes and growth control nodes in low-energy situations to enhance ATP synthesis and decrease ATP consumption [102]. AMPK signalling suppression has a role in the pathophysiology of OA [78]. SIRT1 and SIRT3 are downstream kinases for AMPK. Activation of AMPK-SIRT3 signalling protects cartilage by maintaining the integrity and function of mitochondrial DNA [103]. AMPK and SIRT1 regulate each other, and AMPK can upregulate SIRT1, leading to an increase in autophagy and mitochondrial dysfunction, as well as a decrease in biogenesis, catabolism, and apoptosis [104,105]. Silencing AMPKα2, but not AMPKα1, reduce the expression of SIRT1 in cultured chondrocytes and downregulate autophagy [105]. SIRT1 can also activate the SIRT1/AMPK/peroxisome proliferator-activated receptor γ coactivator-1α (PGC-1α)/peroxisome proliferator-activated receptor gamma (PPAR-γ) pathway, increasing expression of NF-κB, prostaglandin-endoperoxide synthase 2 (COX-2), IL-8, and MMP13, thereby causing dysfunction of chondrocytes and oxidative stress [106].

The function of the AMPK signalling pathway is also correlated with other pathways. For example, Piao et al. found that IL-1β induced chondrocyte inflammation via activation of the AMPK/NF-κB pathway [7]. In addition, activation of the AMPK-mTOR signalling pathway was found to target chondrocyte autophagy and reduce OA inflammation [107]. As a critical bioenergy sensor, AMPK mediates energy homeostasis and mediates the redox balance in chondrocytes to resist various cellular stresses. Abnormal AMPK activity is related to decreased autophagy, impaired mitochondrial function, excessive production of reactive oxygen species, and inflammation of joint tissues [108]. These abnormalities will eventually lead to articular cartilage degeneration, synovial inflammation, and abnormal subchondral bone remodelling. Dynamin-related protein 1 (Drp1) plays an important role in mediating mitochondrial fission in mitochondrial homeostasis [109]. AMPK can activate the AMPK/Drop1 pathway, leading to mitochondrial dysfunction and cell death associated with the opening of the mitochondrial permeability transition pore (mPTP) in chondrocytes (Fig. 4D) [6].

### 4.5. Phosphatase and tensin homologue (PTEN)/ PI3K/AKT signalling pathway in chondrocyte mitochondrial metabolism

PTEN acts as a negative regulator of PI3K/AKT signalling, which is important for cell survival and matrix synthesis [79]. A subsequent decline in PTEN expression/function is related to increased glycolysis and mitochondrial respiration in cells [110]. Expression of PTEN in OA cartilage tissue is significantly upregulated [79]. Reduced expression of PTEN enhances AKT phosphorylation, raises COL2α1 and Aggrecan expression levels, and increases proteoglycan production in response to oxidative stress [111]. Apart from matrix production, the PI3K/AKT signalling pathway is an essential regulator of OA chondrocyte proliferation and differentiation *in vivo* and *in vitro*. In OA, activating the PI3K/AKT signalling pathway promotes chondrocyte growth [112]. To date, there is no report regarding the analysis of PTEN overexpression in chondrocytes (Fig. 4E).

## 5. Novel tools for monitoring chondrocyte bioenergetics

### 5.1. Oxygen consumption rate and Extracellular acidification rate

**Figure 5.**
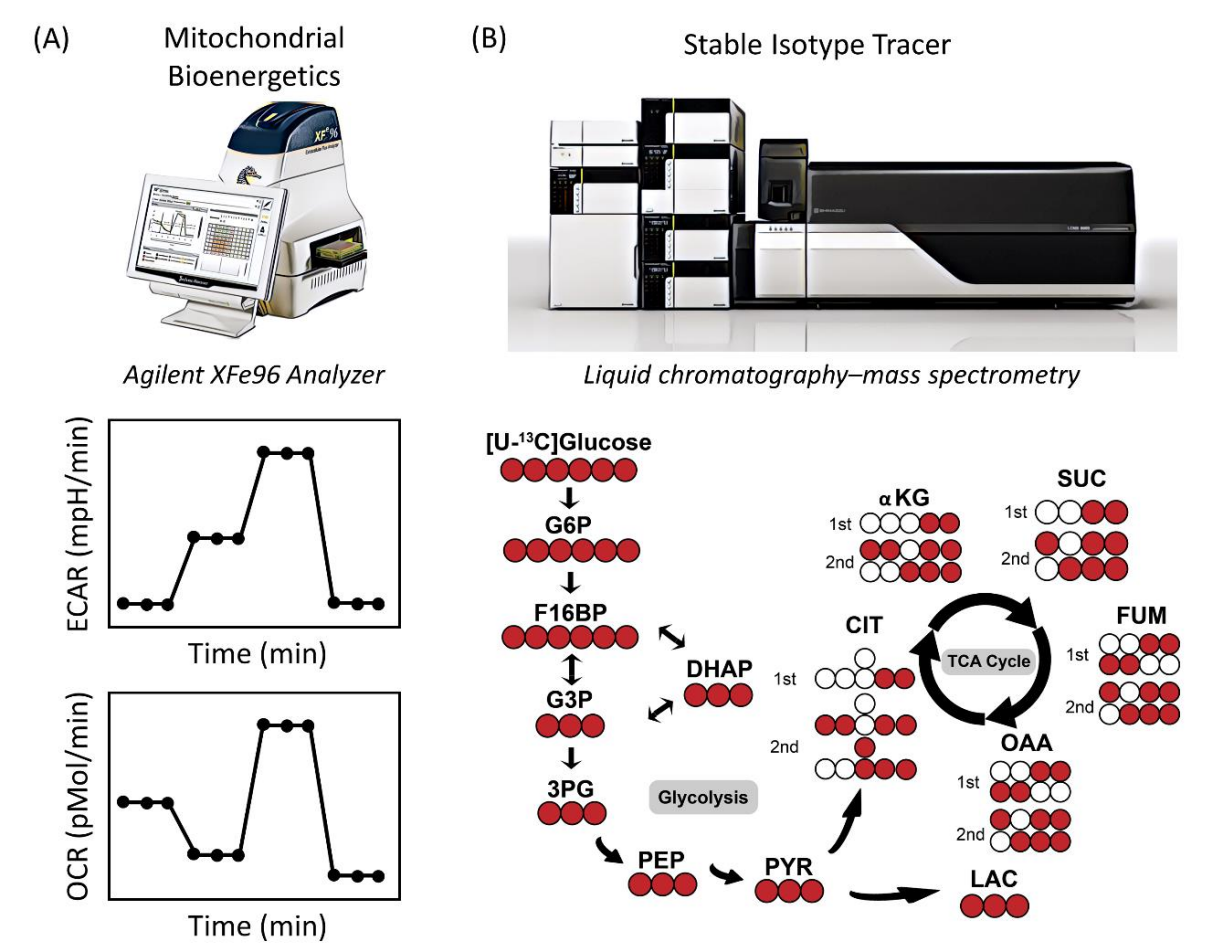
**Novel tools for monitoring chondrocyte bioenergetics**. Schematic overview of the Seahorse metabolic flux assay integration at the biochemical level, LC/MS isotope tracer at the molecular level, and data analysis workflow.

As previously reported, chondrocytes change and adapt in the relatively hypoxic environment in cartilage tissues to sustain their metabolic and bioenergetic requirements. During pathological processes such as OA, chondrocytes change their substrate preference, including increasing glucose metabolism and/or decreasing aerobic respiration [113]. Therefore, the metabolic phenotypes (glycolysis, aerobic or anaerobic) of chondrocytes are different, and measuring or monitoring parameters related to OA markers is of great importance [114]. A greater understanding of chondrocytes metabolic requirements will aid in developing metabolic therapeutics. Two critical factors that connect metabolic reprogramming, metabolic phenotype, and chondrocyte substrate preference, easy-to-measure bioenergetic parameters, are glycolytic function or extracellular acidification rate (ECAR) and mitochondrial respiration or oxygen consumption rate (OCR) [115,116]. To achieve these goals, the Agilent Hippocampal XF analyser is a fast and robust method that can measure OCR and ECAR in cultured cells in real-time and promotes the analysis of cell metabolic activities in a high-throughput manner (Fig. 5). Multiple mitochondrial parameters can be quantified and measured using an XF analyser in conjunction with a Cell Mito Stress assay, such as basic OCR, ATP production, proton leakage, maximum respiratory capacity, and mitochondrial reserve respiratory capacity. Multiple glycolytic parameters can be calculated using the glycolysis stress kit, such as glycolysis, glycolytic capacity, glycolytic reserve, and non-glycolytic acidification (Fig. 5A).

### 5.2. Stable isotope tracer studies

OCR and ECAR provide information about metabolic reprogramming related to glycolysis and mitochondrial respiration. However, studies employing stable isotope tracers using LC/MS provide more quantitative and predictable information regarding changes in glycolysis and TCA intermediates during metabolic reprogramming. The fundamental concepts of stable isotope investigations are summarised below (Fig. 5B) [117,118]. Normal glucose contains six^12^C atoms (atomic weight, 12) (Fig. 5B, white circles). Fully labelled [U-^13^C] glucose (m+6) (Fig. 5B, red circles) was used as a tracer (atomic weight, 13), and glycolytic metabolism was assessed by measuring^13^C_3_-lactate (three^13^C atoms [m+3]). The labelling patterns for TCA cycle intermediates, such as citrate and fumarate, from [U-^13^C] glucose after the first cycle oxidative pathway is [m+2] citrate and [m+2] fumarate, respectively (Fig. 5B). After the second TCA cycle, the labelled intermediates are [m+3] citrate and [m+3] fumarate. Inhibition of the mitochondrial respiratory usually causes a compensatory increase in aerobic glycolysis. Therefore, [U-^13^C] glucose will be broken down into [m+3] pyruvate, followed by a rise in [m+3] lactate in chondrocytes under conditions of impaired respiration.

## 6. Some key puzzles that remain to be solved

The physiological regulation of nutrition and metabolite homoeostasis is required for various fundamental cellular processes in eukaryotic cells, including division, proliferation, differentiation, excitability, secretion, senescence, and death [19]. Each form of metabolic fuel utilized by living cells requires a distinct and well-regulated mechanism of usage that includes transport, regulation, and auxiliary molecules [119]. These findings in cartilage and chondrocytes raise essential questions about nutrient sensing, uptake, storage, and processing mechanisms and the critical roles they play in joint health and disease overall energy homeostasis.

One critical unsolved question is how chondrocyte glycolysis is triggered by OA progression. Is it through feedback regulation of NAD^+^ level or mitochondrial dysfunction? There are three rate-limiting enzymes in the glycolysis pathway, hexokinase, phosphofructokinase-1, and PK [120]. An additional avenue for glycolytic regulation could involve the alteration of these enzymes. Whether abnormal chondrocyte function during OA progression influences these enzymes, which further contributes to the regulation of the chondrocyte glycolysis pathway, is still unclear.

Another question is why chondrocytes activate the glycolysis pathway during OA, and what are the implications of this activation? One molecule of glucose can produce two molecules of ATP through glycolysis while producing 30 molecules of ATP through the TCA cycle and OXPHOS pathway. One possible reason for chondrocytes relying on this less efficient pathway is that glycolysis provides a more rapid energy supply [40]. A similar effect was observed in muscle, where fast-twitch muscle, which requires the highest acute supply of energy, is significantly more glycolytic and contains fewer mitochondria than slow-twitch muscle utilized for postural control [121]. Destruction of cartilage during OA progression stimulates chondrocytes to regenerate extracellular collagen, and perhaps, like fast-twitch muscle fibers, they use glycolysis to ensure rapid resupply of ATP for repair.

Finally, like cancer cells, chondrocytes exhibit the Warburg effect; however, the functional relevance is still unknown. Cancer development is considered a multistep process at the cellular level involving mutations and the gradual selection of cell proliferation, survival, invasion, and metastasis capabilities [122]. Gene mutations can cause cancer by accelerating the rate of cell division or inhibiting the standard control of the system, such as cell cycle arrest or programmed cell death [123]. On the other hand, cartilage is one of the few tissues that cannot be regenerated easily in the human body. Articular cartilage does not have blood vessels, so it cannot regenerate or heal, which means that oxidized red blood cells cannot reach the damaged tissue [124]. However, it has also been reported that the early blood vessels of the subchondral bone in OA can invade calcified cartilage and gradually spread to the superficial cartilage as the disease progresses [125]. In addition, chondrocytes live in an acidic environment, which is also typical of most cancer cells [126,127]. Researchers have discovered that an acidic environment can enhance tumour cells through protein production [122]. When the acidic surface extends beyond the inside of the tumour and comes into contact with healthy tissue, cancer will spread [126]. An increase in glycolysis in chondrocytes contributes to lactate production, which produces an acidic environment in the cartilage [128]. However, the mechanisms that regulate chondrocytes metabolism need further investigation. There are currently no clinical studies focusing on glucose metabolism related metabolic changes in OA cartilage. In the future, more clinical studies and trials are needed to test these ideas regarding chondrocytes, glycolysis, and the Warburg effect in OA.

## 7. Conclusions

In conclusion, emerging evidence indicates the critical role of central carbon metabolism in chondrocyte function during OA progression. Future studies will be required to investigate the comprehensive central carbon metabolism shift in cartilage from normal to OA. It also remains to be determined whether and how the regulation of central carbon metabolism could benefit cartilage health. Thus, a greater understanding of cartilage metabolism may result in a more accurate and timely diagnosis and treatment approach for patients suffering from OA.
